# Impact of timing of follow-up upon outcome in the TOPKAT trial

**DOI:** 10.1186/1745-6215-16-S2-O33

**Published:** 2015-11-16

**Authors:** Jonathan Cook, Graeme MacLennan, David Murray, Andrew Price, Ray Fitzpatrick, Andrew Carr, Marion Campbell, Helen Campbell, Nigel Arden, Cushla Cooper, Loretta Davies, David Beard

**Affiliations:** 1 University of Oxford, Oxford, UK; 2 University of Aberdeen, Aberdeen, UK

## 

Due to the practicalities of recruitment, randomisation of participants in surgical trials may need to take place prior to the day of surgery. However, substantial post-randomisation delays in receiving surgery due to limited capacity may then occur. This can create a tension between timing the follow-up from randomisation (scientifically most desirable) and surgery (clinically most relevant). It is unclear what impact alternative follow-up timings have upon the outcome.

## Methods

TOPKAT compared the clinical and cost effectiveness of total or partial knee replacements for medial compartment osteoarthritis. An additional assessment at 1 year post surgery of the primary outcome measure, Oxford Knee Score (OKS) was administered to patients who had a waiting time following randomisation of greater than 12 weeks as well as baseline and post-randomisation follow-up. Mean difference (MD) between treatments (95% confidence interval (CI)) was calculated using 1 year post-surgery and post-randomisation data.

## Findings

Waiting times for surgery (0-407 days) resulted in a number of participants (n=134 of 531 recruited) receiving their 1 year post randomisation follow up questionnaire at a time point much earlier than 1 year post surgery. However, the mean difference in OKS was very similar for post randomisation (1.8 95% CI (0.2,3.4)) and surgery (1.7 95% CI(0.0,3.3)) analyses.

## Conclusion

Results of the 1 year post-randomisation and surgery follow-ups were very similar. Further assessment in other trials is required to explore the generalisability of this finding. Timing of follow up needs to be carefully chosen to ensure interpretable results.

